# Supplementary material for: Co-construct, implement and evaluate a multi-level intervention to prevent a sedentary lifestyle in children—Study protocol of the CIPRES study
Source: PLoS One. 2024 May 9;19(5):e0302556. doi: 10.1371/journal.pone.0302556 (PMC11081347; doi:10.1371/journal.pone.0302556)
Supplement: Attachment — Submitted filename: Response to Reviewers.docx [file pone.0302556.s001.docx]

**RESPONSE TO REVIEWERS**

**Manuscript Reshape**

In accordance with the PLOS ONE submission guidelines, we have modified the manuscript layout as follows:

- The title format

- The affiliation format

- Removal of keywords

- The format of the three levels of headings

- Removal of text insertion indications for figures

- The format of figure and table titles

- Modification of the insertion of bibliographic references in the text: replacing () with []

- Additional information regarding approval by the ethics committee.

-Bibliography update

- To our knowledge, none of the articles cited in this manuscript have been retracted.
- Line 81 : référence update

Old reference

Anses - Agence nationale de sécurité sanitaire de l’alimentation, de l’environnement et du travail [Internet]. 2016 [cited 2023 Feb 27]. Actualisation des repères du PNNS - Révisions des repères relatifs à l’activité physique et à la sédentarité. Available from: <https://www.anses.fr/fr/content/actualisation-des-rep%C3%A8res-du-pnns-r%C3%A9visions-des-rep%C3%A8res-relatifs-%C3%A0-l%E2%80%99activit%C3%A9-physique-et-%C3%A0>

New reference:

Équipe de surveillance et d’épidémiologie nutritionnelle (Esen). Étude de santé sur l’environnement, la biosurveillance, l’activité physique et la nutrition (Esteban) 2014-2016. Volet Nutrition. Chapitre Activité physique et sédentarité. 2017;Santé Publique France:59.

**Response to reviewers:**

This study protocol covers a cluster RCT of a comprehensive and complex intervention, which requires a disciplinary implementation and data collection. I have two minor comments:

- I checked the study populations in the intervention and control group and wondered how comparable these are as Brignoles is a little town in the rural area, whereas the other areas cover the more urban, coastal areas? The authors could elaborate a little bit more about potential and/or influential differences.

Thank you for your question. Indeed, the town of Brignoles is not a coastal town and has a rural population. The aim of the CIPRES project is to evaluate a sedentary prevention intervention in a wide sample including children from families with heterogeneous social and economic characteristics. To improve representativeness, we chose to characterise our recruitment by taking into account several parameters such as the ecologic deprivation index level (cf. Line 208) and the size of the town (population density). Thus, in order to favour a heterogeneous recruitment, more varied and representative of the Var, we chose to include rural areas (Brignoles), medium-sized towns (La Seyne-sur-Mer, Hyères) and a large town (Toulon) with different levels of EDI ranging from 1 to 5. We will statistically compare the levels of sedentariness in the different areas before the start of the implementation to evaluate if there are any differences according to area and EDI.

- the co-construction of the intervention (line 317 -) is nice but I have little insight in the time frame used> did you start with involvement of parents then followed by teachers and later the community in a fixed time frame of was it at the same time in varying sequence of the stakeholders ? Please clarify

Thank you for your question. As a first step, we identified “gate keepers” in order to make efficient contact with the targeted schools. We then organised meetings bringing together teachers, parents, children, educational advisers and educators in the same time frame. In this way, all the actors involved in the co-construction process were involved at the same time, meeting on a number of occasions.

We added a sentence to clarify this point (cf. line 344-346, revised manuscript with track change): “All the actors were invited to take part in the co-construction at the same time frame, the meetings were spread over several sessions.”
